# Machine learning in image‐based outcome prediction after radiotherapy: A review

**DOI:** 10.1002/acm2.14559

**Published:** 2024-11-18

**Authors:** Xiaohan Yuan, Chaoqiong Ma, Mingzhe Hu, Richard L. J. Qiu, Elahheh Salari, Reema Martini, Xiaofeng Yang

**Affiliations:** ^1^ Department of Biomedical Engineering Emory University and Georgia Institute of Technology Atlanta Georgia USA; ^2^ Department of Radiation Oncology and Winship Cancer Institute Emory University Atlanta Georgia USA; ^3^ Emory School of Medicine Emory University Atlanta Georgia USA

**Keywords:** cancer radiotherapy, machine learning, medical imaging, outcome prediction

## Abstract

The integration of machine learning (ML) with radiotherapy has emerged as a pivotal innovation in outcome prediction, bringing novel insights amid unique challenges. This review comprehensively examines the current scope of ML applications in various treatment contexts, focusing on treatment outcomes such as patient survival, disease recurrence, and treatment‐induced toxicity. It emphasizes the ascending trajectory of research efforts and the prominence of survival analysis as a clinical priority. We analyze the use of several common medical imaging modalities in conjunction with clinical data, highlighting the advantages and complexities inherent in this approach. The research reflects a commitment to advancing patient‐centered care, advocating for expanded research on abdominal and pancreatic cancers. While data collection, patient privacy, standardization, and interpretability present significant challenges, leveraging ML in radiotherapy holds remarkable promise for elevating precision medicine and improving patient care outcomes.

## 1 INTRODUCTION

Radiotherapy is a cornerstone of the multidisciplinary approach to cancer treatment, alongside surgery and chemotherapy. Its evolution from two‐dimensional (2D) conventional radiotherapy to the current state‐of‐the‐art three‐dimensional (3D) digital precision radiotherapy marks a significant leap in personalized cancer care.^1^ This advancement is primarily fueled by the integration of sophisticated imaging technologies—such as computed tomography (CT), magnetic resonance imaging (MRI), positron emission tomography (PET), and ultrasound—into the treatment planning and execution phases. These multi‐modal imaging tools are instrumental in distinguishing tumors from healthy tissue with high precision, enabling radiation oncologists, physicists, and dosimetrists to meticulously tailor and continuously adjust treatment plans. This facilitates maximized therapeutic efficacy while minimizing adverse effects on healthy tissues, thereby enhancing patient outcomes.

Outcome prediction in radiation oncology is essential for clinical decision‐making. It involves assessing a patient's likelihood of survival, cancer recurrence, and potential treatment‐related toxicity. Accurate predictions are crucial for personalized treatment strategies that not only aim to extend life but also preserve or enhance the quality‐of‐life post‐treatment. This necessitates a deep understanding of the disease's behavior and the patient's response to radiotherapy, highlighting the need for advanced predictive models that can effectively analyze and interpret complex clinical data and imaging.

Machine learning (ML), a powerful predictive analytics tool, has revolutionized how outcomes are forecasted across various fields, including healthcare.^2^ This technology leverages statistical methods to enable computers to learn from and make predictions or decisions based on data. In radiation oncology, ML's ability to analyze complex datasets and identify hidden patterns has led to significant advancements in outcome prediction. This process has been facilitated by the growth in computational power and the availability of large datasets, allowing for more accurate and nuanced predictions. In recent years, there has been a transformative development in ML technologies,^3^ significantly impacting the field of outcome prediction in radiation oncology. The transition from traditional ML techniques to more sophisticated deep learning (DL) models exemplifies this shift. DL, a subset of ML, excels in handling vast arrays of clinical data and medical images, enabling the extraction of intricate patterns and features that were previously unattainable. This capability has dramatically improved the accuracy and comprehensiveness of outcome predictions, facilitating the shift towards more individualized treatment plans. The integration of DL models with diverse imaging data—such as PET, CT, MR, and ultrasound—has fortified the role of ML in enhancing decision‐support tools,^4^ thereby making a significant stride toward achieving personalized medicine in cancer treatment. CT imaging, known for its detailed cross‐sectional views, is pivotal in visualizing anatomical structures with high resolution. MRI provides valuable insights into tumor morphology and surrounding anatomical structures with its exceptional soft tissue contrast and multi‐planar capabilities. PET imaging, usually combined with a CT scan, offers functional and metabolic information alongside anatomical details. Ultrasound imaging is specialized in real‐time visualization of soft tissues. Different imaging types, each with its unique specialties, play a crucial role in outcome prediction in radiotherapy.

Despite advancements, ML in radiotherapy faces significant challenges including privacy concerns, data sharing limitations, and the extensive time needed to collect sufficient patient data. Additional hurdles like data standardization and the interpretability of complex models complicate their effective use.^5^ These issues, which will be discussed further in this paper, must be addressed to fully harness ML's potential in improving cancer treatment.

The field continues to grow rapidly, driven by clinical needs and the potential of these advanced technologies to improve patient outcomes. ML has increasingly become a prominent force within medical imaging, attracting significant contributions from many researchers dedicated to enhancing this field.^6^ A wealth of impactful studies has explored the integration of ML techniques in medical imaging, focusing on their application in diagnosis, treatment planning, and more.

In this review, we explore the integration of medical imaging technologies and ML in enhancing outcome predictions in radiotherapy. The forthcoming sections will delve into the theoretical basics of these technologies, followed by a detailed examination of their practical applications across various cancer treatment sites. By assessing the impact of advanced imaging modalities and ML algorithms on patient survival, disease recurrence, and treatment‐related toxicities, this manuscript aims to highlight the pivotal role of these technologies in refining radiotherapy practices. Furthermore, we will analyze the current challenges and propose future directions for research, underscoring the potential of these integrated approaches to revolutionize personalized cancer therapy and improve patient care outcomes.

## 2 LITERATURE SEARCH

This review paper investigates the use of ML in radiotherapy, particularly in the domain of outcome prediction. The initial literature search, conducted on PubMed, was based on a combination of keywords: “outcome prediction”, “medical imaging”, “machine learning”, and “cancer radiotherapy”. To ensure an exhaustive coverage of the relevant research, the search criteria were refined by replacing “medical imaging” with specific imaging techniques, namely PET, CT, MR, and ultrasound.

This comprehensive review involved a rigorous screening of relevant publications, focusing on those that matched the search criteria from 2010 through July 2024. The initial search revealed a notable emergence of relevant papers beginning in 2017, with a significant increase in publications. After a thorough evaluation, 85 articles were selected for their direct relevance and contribution to the study's objective.

**FIGURE 1:**
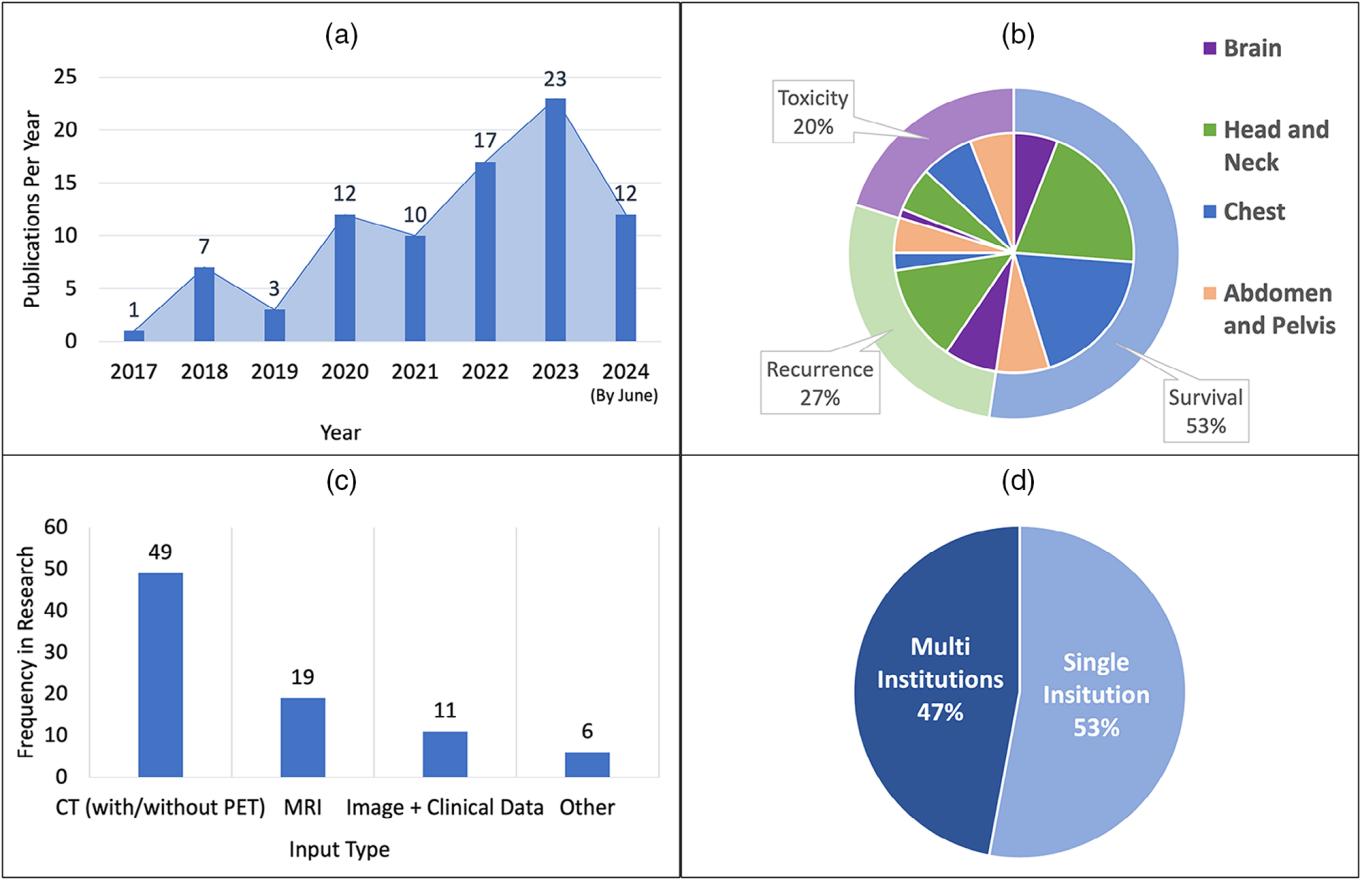
(a) Trend analysis of annual publications; (b) distribution of outcome prediction categories by treatment site; (c) prevalence of imaging modalities in research, which includes image + clinical data (models using MRI or CT combined with clinical information), multi‐image types (models using more than one type of image), and other (images such as immunohistochemistry images, pathological microscopic features, etc.); (d) institutional collaboration in data sourcing.

Figure 1 provides a summary analysis that synthesizes findings from selected papers, offering insights into research trends, predictive outcomes, imaging techniques, and trends in data sourcing within the medical field. The literature review reveals a consistent rise in research efforts, particularly notable post‐2020. This notable trend in publication volume, with most directly related papers emerging in the past 3 years, underscores the rapidly growing importance and recognition of ML techniques in the context of enhancing treatment outcomes in radiotherapy. Figure 1a illustrates a linear increase in the number of annual publications, indicating a growing reliance on ML in oncological applications. In terms of research focus, as shown in Figure 1b, survival prediction dominates, accounting for 53% of studies. This is followed by recurrence and toxicity prediction, at 27% and 20%, respectively, highlighting the breadth of patient outcomes being addressed. Furthermore, the prevalence of CT scans in studies is evident in Figure 1c, with a significant portion of research also utilizing MRI, demonstrating the clinical preference for detailed imaging in outcome prediction. There is also a noteworthy trend towards integrating imaging with clinical data, aiming to leverage the combined strengths of both data types. Lastly, Figure 1d shows that over half of the studies are based on data from single institutions, which poses certain limitations on data diversity. Conversely, 47% of studies using multi‐institutional datasets indicate a movement towards broader data inclusion to enhance model robustness.

## 3 BASICS OF RADIOTHERAPY, OUTCOME PREDICTION, MACHINE LEARNING, AND EVALUATION METRICS

### 3.1 Radiotherapy

Radiotherapy employs ionizing radiation, such as x‐rays and gamma rays, to effectively manage cancer in both curative and palliative settings. Rather than directly altering cellular structures, these high‐energy photon rays primarily interact with the water molecules within cells to produce free radicals. These free radicals then cause ionization and excitation that lead to DNA damage in cancer cells, thus inhibiting their ability to proliferate and metastasize. In this review, collected papers included several different types of radiotherapy, such as external beam radiotherapy (EBRT) and conformal radiotherapy (CRT). Each of these employs specific technologies and radiation types.

Radiotherapy requires precision to ensure the delivery of maximal therapeutic doses to malignant tissues while minimizing exposure to surrounding healthy cells. Within this framework, radiomics significantly enhances the capabilities of radiotherapy by providing a more detailed and quantifiable analysis of tumor characteristics.

Radiomics, an innovative approach within radiotherapy, extracts a vast array of quantitative data from standard medical images. This method captures detailed tumor characteristics, such as heterogeneity, morphology, and textural patterns, that go beyond what is discernible through conventional imaging techniques. For instance, by analyzing textural patterns on CT scans, radiomics can predict which tumors are more likely to respond to radiation therapy based on their microenvironment characteristics, thus transcending traditional imaging barriers. By integrating radiomics, clinicians can achieve a more personalized treatment strategy.

### 3.2 Outcome prediction

This review paper focuses on elucidating the role of ML in forecasting three critical aspects of radiotherapy outcomes: patient survival, disease recurrence, and treatment‐related toxicity.

#### 3.2.1 Survival prediction

Survival prediction in radiotherapy refers to the estimation of a patient's likelihood of surviving following treatment. This prognosis is pivotal in treatment planning and patient counseling.

Overall survival is the most straightforward and commonly used measure. It denotes the duration from the start of treatment until death from any cause. It provides a holistic view of treatment efficacy. Progression‐free survival measures the time during which a patient lives without any signs of cancer progression. This metric is crucial for understanding the effectiveness of radiotherapy in halting disease advancement.

#### 3.2.2 Recurrence prediction

Cancer recurrence, also known as cancer relapse, is the reappearance of cancer after treatment and may occur in the same or different part of the body. In radiation oncology, it is critical to predict the likelihood of cancer reappearing at the original site (local recurrence) or elsewhere in the body (distant recurrence).

Local control/failure specifically refers to the success or failure of radiotherapy in preventing the cancer from returning to the initial treatment site.

#### 3.2.3 Toxicity prediction

In cancer radiotherapy, toxicity prediction involves estimating the adverse effects caused by the treatment. These effects are broader than direct treatment damage. Toxicity in this context includes both short‐term toxicity and long‐term consequences that may arise post‐treatment, affecting the patient's life quality. An appropriate example is radiation pneumonitis caused by radiotherapy to the lung. This condition, characterized by inflammation of the lungs, is a significant concern and falls under the umbrella of toxicity.

### 3.3 Machine learning

#### 3.3.1 Methodologies

ML, as a key innovation within artificial intelligence, has revolutionized numerous fields, including radiotherapy in cancer care. It is broadly divided into three categories: supervised, unsupervised, and reinforcement learning, with supervised learning being particularly crucial for enhancing radiotherapy treatments. This technology employs algorithms to model and understand complex patterns in data, facilitating more accurate predictions and decisions without explicit programming. ML can be broadly categorized into two main types: traditional ML and DL.

Traditional ML methods, which we refer to as non‐DL methods, include techniques such as regression, classification, clustering, and dimensionality reduction. These methods typically require manual feature engineering, where significant domain knowledge is necessary to identify and select the most relevant features from the data before model training. Examples include support vector machines (SVM), decision trees (DT), and random forests (RF),^7^ which rely on handcrafted features for model training. They have been implemented in classifying patient data, predicting treatment outcomes, and assessing the likelihood of disease recurrence or radiation‐induced toxicity. The strength of traditional ML lies in its interpretability and the ability to incorporate domain‐specific knowledge into the feature engineering process, making it a valuable tool for initial explorations in outcome prediction and treatment optimization.

DL, on the other hand, utilizes more complex algorithms based on artificial neural networks with multiple layers (hence “deep”). These networks, inspired by the neural architecture of the human brain, excel in handling vast amounts of data and learning complex patterns through multiple layers of abstraction. DL methods, especially convolutional neural networks (CNNs) for image analysis,^7^ outshine traditional techniques by automatically extracting relevant features from medical images, eliminating the need for manual feature extraction.

The latest advancements in ML for radiotherapy encompass hybrid models that combine the strengths of traditional algorithms with the DL paradigm. These approaches leverage the interpretability of traditional models and the feature representation power of DL to provide a more nuanced understanding of cancer treatment outcomes. By integrating different types of data, including imaging, genomic, and clinical records, hybrid models offer a comprehensive view of patient prognosis, enhancing the precision of treatment plans.

In the domain of outcome prediction post‐radiotherapy, researchers employ ML through three primary methodologies:

Traditional ML with Feature Engineering: This approach^8^ combines traditional ML algorithms (like SVM, DT, or RF) with handcrafted features (based on domain knowledge) and/or deep‐learned features (extracted from DL models). This method benefits from the interpretability of traditional ML models and the powerful feature representation capabilities of DL.

For example, SVM is a commonly used traditional ML algorithm^9^ renowned for its ability to identify the optimal boundary, or hyperplane, that separates different classes within a feature space. This technique is particularly potent in medical settings for categorizing outcome scenarios, such as patient response to treatments. In addition to SVM, Random Survival Forests (RSF) is another popularly used algorithm tailored specifically for survival data analysis as an extension of the classic RF algorithm.^10^ This method builds an ensemble of DT, each trained on a randomly selected subset of data, and focuses on predicting survival outcomes.

Deep learning‐only approach: This method exclusively uses DL techniques, primarily focusing on image analysis.^11^, ^12^ The process typically involves image preprocessing, feature extraction through deep neural networks (like CNNs), and model training for prediction tasks. This approach leverages the ability of DL to automatically learn complex features from medical images, which is crucial for accurate outcome prediction.

For instance, 3D‐CNNs,^13^ a variation of the traditional CNN, are specifically designed to analyze 3D data. In the context of radiotherapy, they are commonly used for processing 3D medical images, such as CT or MRI scans. By extending the convolutional layers to three dimensions, 3D‐CNNs can capture spatial hierarchies and patterns in volumetric data.

Hybrid deep learning approach: This method combines DL techniques with traditional ML algorithms to leverage the strengths of both approaches. For example, it might integrate CNNs for image feature extraction with traditional statistical models to analyze sequential data like treatment histories. This hybrid approach is designed to capture both spatial (from images) and temporal (from treatment data) dimensions, providing a holistic analysis that enhances the understanding of complex treatment outcomes.

Within outcome prediction, transfer learning emerges as a particularly valuable strategy, especially given the unique challenges associated with acquiring large‐scale datasets in this domain. Unlike applications focused on diagnosis, where extensive image libraries are more commonly available, the datasets required for accurate outcome prediction are often smaller due to the specificity of the data and the complexity of obtaining long‐term patient follow‐ups. Additionally, in this context, data augmentation techniques—commonly used to artificially expand training datasets—may not be suitable due to the risk of introducing bias or inaccuracies that could significantly affect the predictive integrity of the models. Therefore, transfer learning, which involves adapting pre‐trained models developed on large, diverse datasets to specific, smaller datasets, offers a compelling solution. This approach leverages the generalized feature‐detection capabilities acquired from broader imaging tasks, enhancing model performance without the need for extensive native data. Establishing large inter‐institutional radiology and clinical databanks would further amplify the benefits of this approach, enabling more robust generalization across diverse patient populations and clinical settings.

#### 3.3.2 Workflow in outcome prediction

**FIGURE 2:**
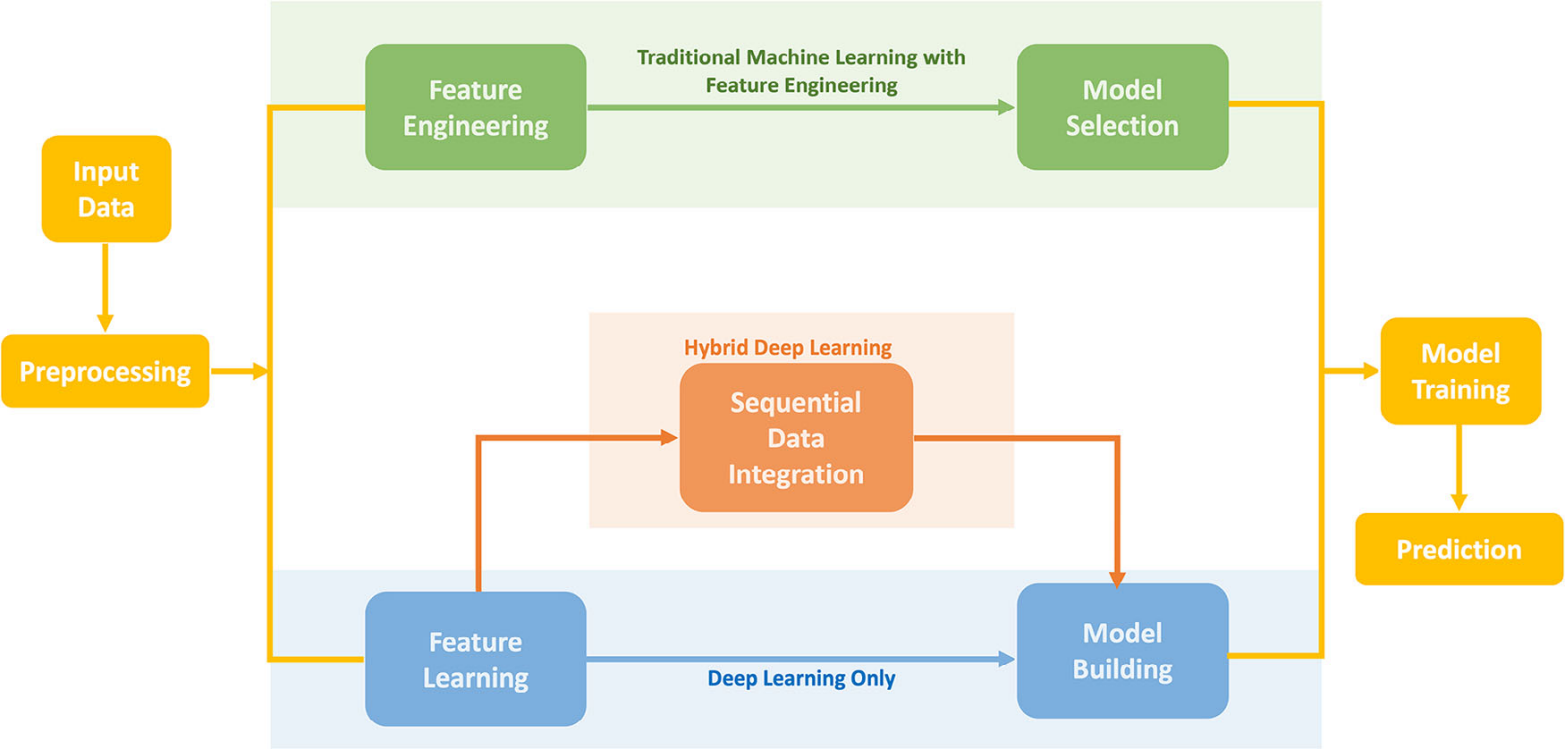
The spectrum of machine learning methodologies.

As shown in Figure 2, the journey of all three methodologies—Traditional ML with Feature Engineering, Deep Learning‐Only, and Hybrid Deep Learning—begins with “Input Data”. This is the raw medical imaging data, such as MRI or CT scans, which forms the substrate for analysis. Some studies also incorporated clinical data alongside images to enhance the scope of their research. Following this, “Preprocessing” is a universal step, where data is prepared for further analysis. This might include noise reduction, normalization, or augmentation to ensure that the input data is clean and consistent. After preprocessing, each methodology diverges according to its unique approach to handling data. However, they reconvene at the “Model Training” stage, where the respective models learn from the preprocessed data or extracted features. The final step in the shared workflow is “Prediction”, where the trained and validated models are finally applied to new, unseen medical images and/or clinical data to predict outcomes.

Traditional ML with Feature Engineering is distinguished by its reliance on domain expertise at the “Feature Engineering” step. Here, insightful features are manually crafted from the imaging data. Feature engineering primarily involves two key processes: (1) Feature extraction, which includes deriving radiomics features from images and clinical data, and (2) feature selection, aimed at identifying and retaining the most informative features for analysis. These features might encapsulate specific patterns or textures recognized as diagnostically significant by medical professionals. Following feature engineering, “Model Selection” is pivotal, as it involves choosing an algorithm—like SVMs, DTs, or RFs—that best fits the manually engineered features. In contrast, the Deep Learning‐only approach bypasses manual feature engineering altogether, instead employing “Feature Learning” where deep neural networks, particularly CNNs, automatically learn to identify intricate patterns in the data. The “Model Building” step for DL is integral to the feature learning process and typically involves setting up layers and structures of the neural network. Hybrid DL merges the strengths of both worlds. It incorporates “Sequential Data Integration” after feature learning, using techniques like RNN or long short‐term memory (LSTM) models to assimilate temporal or sequential data that might be pertinent to patient histories or treatment progression.

### 3.4 Evaluation metrics

Accurately assessing the performance of predictive models is crucial to ensure reliability and efficacy. Accuracy, the receiver operating characteristic (ROC) curve, the area under the curve (AUC), and concordance index (c‐index) are commonly used metrics to evaluate model performance in medical imaging and radiotherapy outcome prediction. Accuracy quantifies the overall effectiveness of a model by measuring the proportion of true results (both true positives and true negatives) in the total population, with higher numbers denoting improved performance. The ROC curve is a graphical plot that illustrates the diagnostic ability of a classifier by plotting the true positive rate (sensitivity) against the false positive rate (1 – specificity) at various threshold settings. The AUC provides a numerical summary of the ROC curve, with a higher AUC (approaching 1.0) denoting a better model. C‐index measures the ability of a model to correctly rank the predicted outcomes with respect to actual outcomes, with a value of 1.0 indicating a perfect model.

While many other metrics could be employed depending on the specific requirements and contexts of the study, these metrics are commonly used due to their interpretability and the comprehensive insight they provide into model performance. Employing a range of evaluation metrics helps in thoroughly understanding a model's strengths and weaknesses. In this paper, for those collected studies that contain these evaluation metrics, we have organized and presented them in the tables, allowing for a clear view of the models' performances across different studies.

## 4 APPLICATIONS IN DIFFERENT TREATMENT SITES

### 4.1 Brain

**FIGURE 3:**
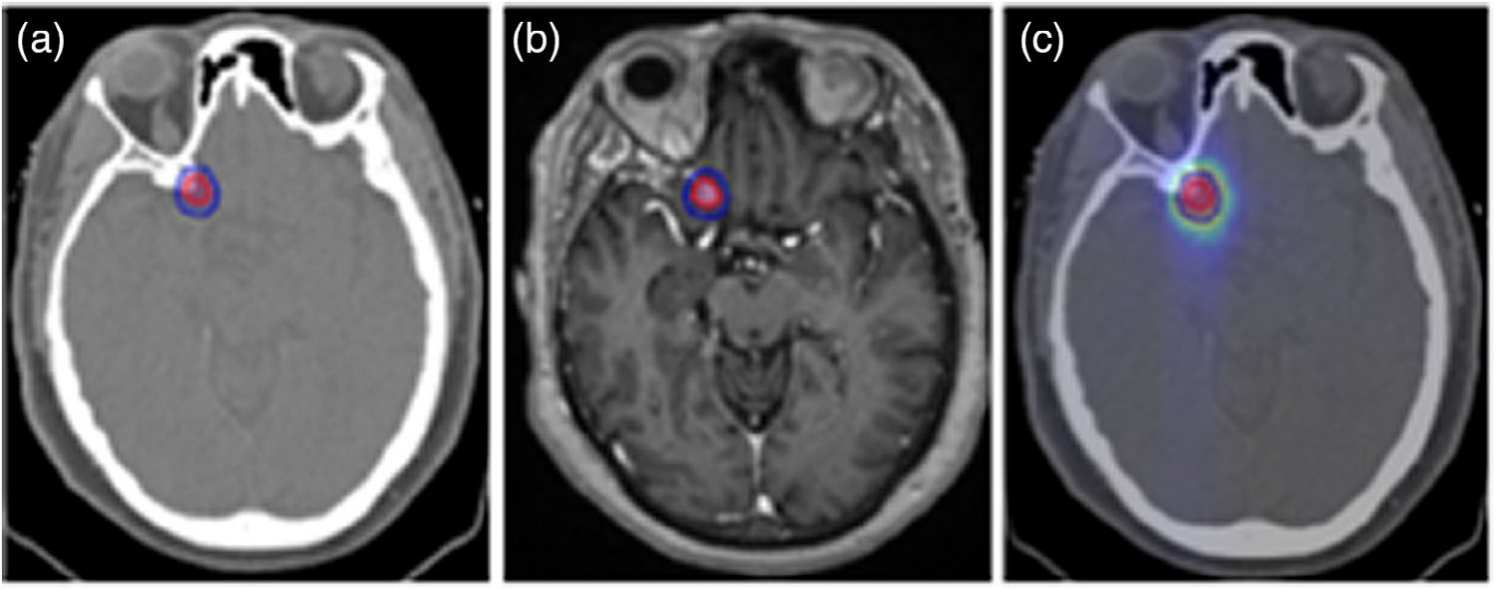
Right clinoid meningioma visualization in a 74‐year‐old female patient. Images procured from our institution. (a) Axial view of the planning CT showing the tumor with delineated clinical target volume (CTV) and planning target volume (PTV) in velocity software. (b) Axial view of the MRI (T1 MPRAGE) in velocity illustrating the CTV and PTV contours. (c) Axial view of the planning CT highlighting the tumor contours (CTV and PTV) and a dose color wash, dose window level of 5 to 45 Gy.

Table 1 lists all the studies on the outcome predictions for brain cancers. Among these 13 studies focusing on the application of ML for outcome prediction in the brain, 6 papers addressed survival outcomes, 6 focused on recurrence/local failure/local control, and 1 concerned toxicity‐related outcomes. These studies mainly focused on cases of glioblastoma and brain metastases. An illustrative case is presented in Figure 3, displaying the planning CT and MRI images, along with the dose distribution for a case of right clinoid meningioma in a 74‐year‐old female patient who received radiotherapy at our institute. Medical images, such as CT, MRI, and PET scans, serve as the input data for ML algorithms to predict treatment outcomes.

**TABLE 1:** Survey of brain cancers.

Outcome	Reference	Tumor type	Category	Image modality	Sample size	Database source	Major method	Prediction performance
Survival	14	Glioblastoma	Survival at 8 months post‐radiotherapy	MRI	206	11 Institutes	Modified DenseNet121	Best AUC = 0.93 for imaging model on amalgamated test set
	15	Lung Cancer Brain Metastases	Overall survival	MRI, and clinical information	237	1 Institute	DeepSurv	c‐index = 0.75, AUC = 0.82, 0.80, 0.84, and 0.92 for predicting survival status at 3, 6, 12, and 24 months
	16	Pediatric Medulloblastoma	Survival	MRI	253	2 Institutes	Cox regression with the LASSO	c‐index = 0.711 (training), 0.707 (test sets 1), 0.717 (test sets 2)
	17	High‐Grade Glioma	Survival	CT	95	1 Institute	RSF, SVM, and CPHs	RSF: c‐index = 0.824 and 0.847. SVM: c‐index = 0.792 and 0.823. CPH: c‐index = 0.821(training) and 0.811 (testing). AUCs = 92.4%, 87.7%, and 84.0% for 1‐, 2‐, and 3‐year survival.
	18	Glioblastoma	Survival	CT	260	Multiple institutes open dataset	RSF and CoxPH models with Bayesian hyperparameter optimization.	Best c‐index = 0.823
	19	Brain Metastases	Survival	CT	1673	Multiple institutes open dataset	DeepSurv, CoxPH and RSF	Best c‐index = 0.7488 with DeepSurv
Recurrence	20	Brain Metastasis	Local failure	MRI	124	Multiple institutes open dataset	3D deep ResNets,	Best AUC = 0.88 to 0.91 for 3D residual network with self‐attention on test set
	21	Glioblastoma	Recurrence	MRI	6	2 Institutes	CNN	Positive, statistical significance cannot be established.
	22	Brain Metastasis	Local control/failure	MRI	124	Multiple institutes open dataset	InceptionResnetV2	Best AUC = 0.86
	23	Brain Metastasis	Local failure	CT	120	Multiple institutes open dataset	Bagging with DT, KNN, AdaBoost with DT	Best accuracy = 71% and AUC = 0.72 on an independent test set
	24	Brain Metastases	Local failure (LF)	MRI	100	Multiple institutes open dataset	SVM	AUC = 0.80, 0.81, and 0.79 for the 6‐month, 12‐month and overall LF
	25	Brain Metastasis	Local control/failure	MRI	38	Multiple institutes open dataset	SVM	AUC = 0.8, accuracy = 82%
Toxicity	26	Brain Arteriovenous Malformation	Favorable outcomes (obliteration without complication)	MRI	130	1 Institute	SVM	Best AUC = 0.78 (training), 0.77 (testing)

Abbreviations: AdaBoost, adaptive boosting; CoxPH, Cox proportional hazards model; CNN, convolutional neural network; DeepSurv, a deep learning model adapted from Cox proportional hazards regression; DenseNet, densely connected convolutional network; DT, decision tree; Inception‐ResNet‐v2, a convolutional neural network; KNN, k‐nearest neighbor algorithm; LASSO, least absolute shrinkage and selection operator; ResNet, residual network; RSF, random survival forest; SVM, support vector machine.

The accuracy of these studies' predictions varies, showcasing the variety and complexity of the methods used. Most of the studies assessed prediction performance using the AUC and c‐index, with larger values indicating better performance. Specifically, in Table 1, nine studies report their results using AUC, and five utilize the c‐index. The reported AUC for survival outcomes spans from 0.80 to 0.93, with the highest accuracy reported by Chelliah et al. (2024).^14^ The research centered on recurrence or local control produced AUCs ranging from 0.72 to 0.91, with the study by Jalalifar et al. (2023)^20^ leading in accuracy. For the study on toxicity, the AUC was 0.78 during training and 0.77 during testing, as reported by Meng et al. (2022).^26^

Taking a closer look at recurrence, including local control and failure, earlier studies mainly utilized traditional ML techniques, notably the work of Karami, Soliman, Ruschin, et al. (2019).^24^, ^25^ These studies commonly employed SVMs and reported fair performance with AUCs around 0.8. A shift is observed in more recent research, with several studies moving towards DL models and implementing CNNs as their primary method, as seen in the work of Jalalifar et al. (2022),^22^ and Pouessel et al. (2023)^21^ (Table 1). This transition signals a promising trend in the evolution of predictive studies, similar to those focusing on survival outcomes. This transition marks a significant evolution from relatively simpler, traditional ML models to more complex and sophisticated DL architectures. This shift reflects a broader trend toward leveraging advanced computational techniques to enhance predictive accuracy in survival outcomes across various studies. For instance, the use of CNN in analyzing medical images has improved the ability to identify subtle patterns associated with long‐term survival, which traditional models might overlook.

### 4.2 Head and neck

**FIGURE 4:**
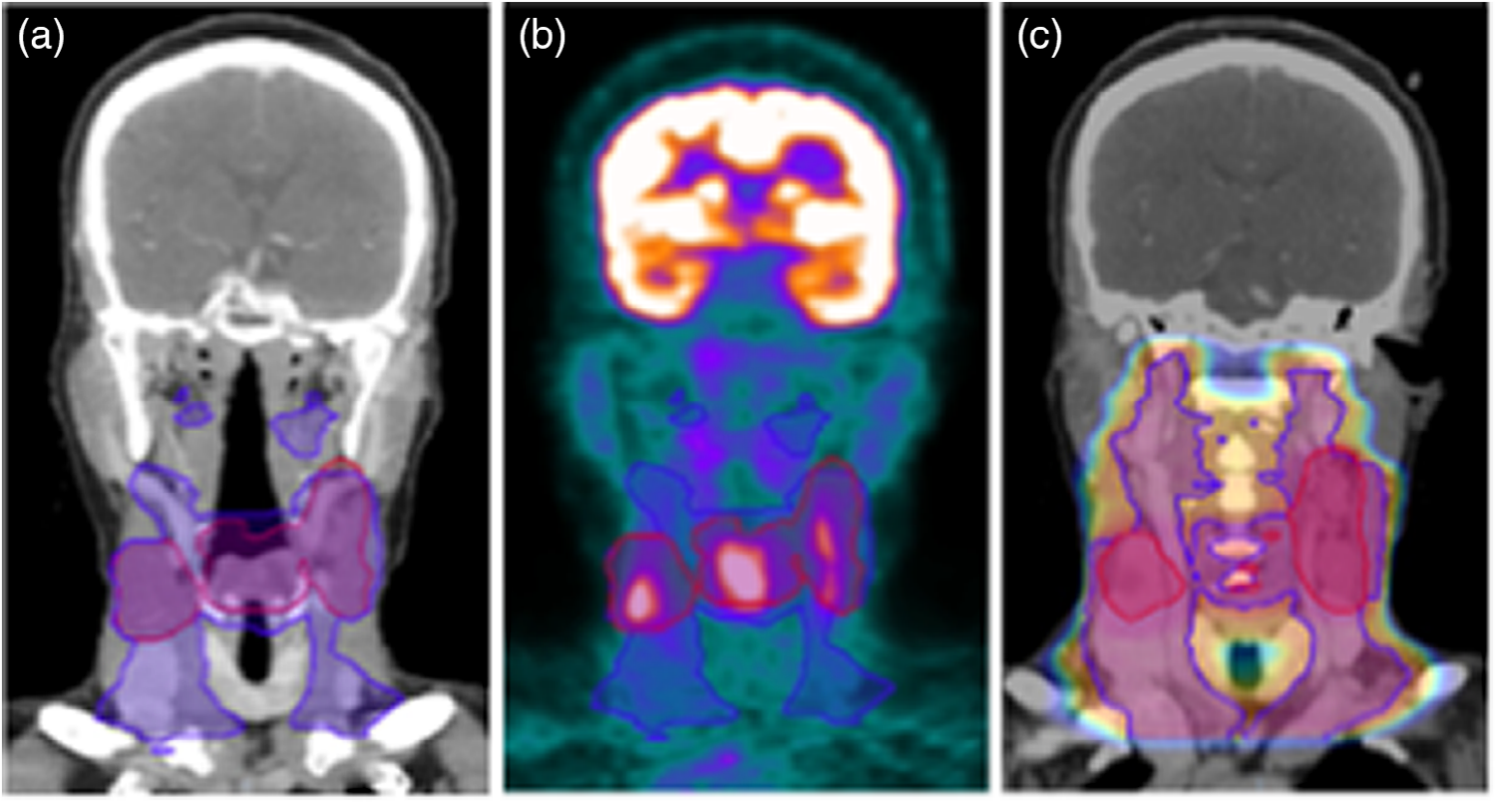
Imaging of a Stage T3N3b squamous cell carcinoma of the glottic larynx in a 70‐year‐old female procured from our institution. (a) Coronal view of the planning CT with clinical target volumes (CTV 7000 and CTV 6300) outlined. (b) Coronal view of the PET scan showing CTV 7000 and CTV 6300, with a window level of 0 to 8 standard uptake value (SUV). (c) Coronal view of the planning CT image with CTV 7000 and CTV 6300 contours, augmented with a dose color wash: dose window level set from 28 to 70 Gy.

Head and neck cancer refers to cancers that develop in the mouth, nose, sinuses, salivary glands, throat, and larynx. Across 30 studies focusing on H&N cancers, diverse subtypes such as esophageal squamous cell carcinoma, oropharyngeal cancer, and nasopharyngeal carcinoma were included, among cancer types. Eighteen papers addressed survival outcomes, 12 focused on recurrence/local failure/local control, and 7 concerned toxicity‐related outcomes. Notably, the total counts of studies focusing on specific outcomes exceed 30 because 7 papers tackled multiple outcomes. While many studies focus on a single aspect of patient outcomes, several others examine multiple outcomes within the same research. For example, studies by De Biase et al. (2024),^27^ Ma et al. (2023),^28^ Le et al. (2022),^29^ Pang et al. (2022),^30^ Fh et al. (2021),^31^ and Folkert et al. (2017)^32^ assessed both survival and recurrence, whereas another might analyze survival and toxicity as demonstrated by Bogowicz et al. (2020).^33^ For visual insight, Figure 4 presents imaging of a Stage T3N3b squamous cell carcinoma of the glottic larynx in a 70‐year‐old female, presenting the complex planning involved in targeting such cancers. Each paper's contribution is recorded under each relevant outcome category. Detailed information on these studies can be found in Table 2. This rule is consistent across the following studies in other treatment areas, highlighting an increasing recognition of the importance to consider not only patient survival but also the quality‐of‐life post‐treatment.

**TABLE 2:** Survey of head and neck cancers.

Outcome	Reference	Tumor type	Category	Image modality	Sample size	Database source	Major method	Prediction performance
Survival	34	Head and Neck Cancer	Overall survival	CT and 3D dose maps	240	Multiple institutes open dataset	CoxBoost, RSF, CoxPH, glmboost, glmnet, and ST	Best c‐index = 0.73 with CoxBoost
	35	Head and Neck Squamous Cell Carcinoma	Survival	CT and EMR data	3425	3 Institutes	Multitask learning	High accuracy for 2‐year and lifetime survival prediction. Performance decreased in external datasets
	36	Oropharyngeal Cancer	Survival	CT	427	Multiple institutes open dataset	ML with LASSO and SFBS, XGBoost with SHAP	AUC = 0.85
	37	Nasopharyngeal Carcinoma	Survival	MRI	151	1 Institute	DL LR	Highest AUC = 0.88 of DL model
	38	Metastatic Neck Lymph Nodes	Overall survival	PET/CT	79	1 Institute	Machine learning model using XGBoost algorithm	Original dataset performance: Accuracy = 0.76 Over‐sampling: Accuracy = 0.80 Under‐sampling: Accuracy = 0.71
	39	Oropharyngeal Squamous Cell Carcinoma	Survival	PET/CT	652	Multiple institutes open dataset	CNN	Best c‐index = 0.787
	40	Nasopharyngeal Carcinoma	Survival	MRI	1872	4 Institutes	SE‐ResNet	c‐index = 0.784–0.921
	41	Head and Neck Squamous Cell Carcinoma	Overall survival	Not Specified	33,527	Multiple institutes open dataset	DeepSurv, N‐MLTR, and RSF	Hazard ratio of 0.79 for DeepSurv, showing survival benefit
	42	Nasopharyngeal Carcinoma	Survival	Pathological microscopic features.	1055	1 Institute	DeepSurv	c‐index = 0.723; high‐risk group showed shorter 5‐year PFS
	43	Head‐and‐Neck Cancer, specifically oral tongue area	Survival	CT	59	1 Institute	BP, GA‐BP and PGA‐BP neural networks.	PGA‐BP yielded the best predictive performance. Predicted survival intervals of 35.8 ± 15.2, 32.3 ± 13.1, and 31.6 ± 15.8 months for BP, GA‐BP, and PGA‐BP models
	44	Nasopharyngeal Carcinoma	Survival	MRI	638	1 Institute	SE‐ResNeXt	c‐index = 0.695–0.731 (training) and 0.706–0.755 (test)
Recurrence	45	Ocular Adnexal Lymphoma	Incomplete remission after radiotherapy	MRI	87	1 Institute	SVM, XGBoost, Lasso, RF and LR	Best AUC = 0.80 with Lasso and RF intersection for ML methods
	46	Oesophageal Squamous cell carcinoma	Local response	PET/CT	142	1 Institute	DT, SVM, KNN, and neural network classifiers	Best accuracy = 84.4% for CT‐based radiomics based on the KNN classifier, 86.0% for PET‐based radiomics, and 79.0% for dosiomics based on the NN classifiers
	47	Head and Neck Squamous Cell Carcinoma	Distant metastases, locoregional recurrence, new primary, and residual disease	CT	311	1 Institute	RFM, KSVM and XGBoost	Accuracy for Distant Metastases, Locoregional Recurrence, New Primary, and Residual Disease = 97%, 72%, 99%, and 96%
	48	Laryngeal and Hypopharyngeal Cancer	Local recurrence	MRI	70	1 Institute	CNN	AUC = 0.767, accuracy = 81.0%
	49	Locally Advanced Head and Neck Squamous Cell Carcinoma	Loco‐regional tumor control	CT	291	Multiple institutes	3D‐ and 2D‐CNN	Best c‐index = 0.31 with 3D‐CNN ensemble
	50	Head and Neck Cancer	Locoregional failure	CT	190	1 Institute	DL‐CNN	Highest precision recall AUC = 0.66
Toxicity	51	Lung Cancer	Radiation pneumonitis	CT, clinical, dosimetric, and laboratory data	548	1 Institute	MergeNet, SVM, LGBM and convolution‐only neural network	AUC = 0.689, 0.525, 0.541 and 0.550 for MergeNet, SVM, LGBM and convolution‐only neural network, respectively
	52	Head and Neck Cancer	Late patient‐reported dysphagia	CT	87	1 Institute	RF	Accuracy = 0.71 (validation), 0.73 (testing)
	53	Locally Recurrent Nasopharyngeal Carcinoma	Post‐radiation nasopharyngeal necrosis	MRI	761	4 Institutes	RF	AUC = 0.713–0.756
	54	Nasopharyngeal Carcinoma	Nasopharyngeal necrosis	MRI	230	1 Institute	LR, RF, SVM, and Adaboost combined with multi‐modal information fusion	Best AUC, ACC, SEN, and SPE = 0.936, 0.85, 0.857, and 0.692, respectively
	55	Nasopharyngeal Carcinoma	Radiation necrosis occurrence	MRI	74	1 Institute	Multiple ML methods	AUC at 1–3 months post‐radiotherapy = 0.879 AUC at 6 months post‐radiotherapy = 0.806 AUC for predictive model with absolute volume at 1–3 months: 0.842
	56	Head and Neck Cancers	Sensorineural hearing loss	CT	47	1 Institute	ML methods and LASSO penalized LR	More than 70% predictive power (accuracy, precision, AUC)
Combined	27	Oropharyngeal cancer	Local control, regional control and overall survival	PET/CT	399	1 Institute	3D ResNet18	Best c‐index = 0.74 for local control and 0.60 for regional control, and 0.74 for overall survival
	28	Oropharyngeal Squamous Cell Carcinoma	Local control, regional control, locoregional control, distant metastasis‐free survival, tumor‐specific survival, overall survival and disease‐free survival	CT	606	1 Institute	CoxPH	c‐index = 0.91 in RC, 0.74 in DMFS, 0.82 in LC
	29	Head and Neck Squamous Cell Carcinoma	Distant metastasis, locoregional recurrence, overall survival.	PET/CT	669	Multiple institutes open dataset	PreSANet with pseudo‐volumetric CNN	AUROC = 80%, 80%, and 82% for distant metastasis, locoregional recurrence, and overall survival respectively on the public dataset
	30	Head and Neck Cancer	Distant metastasis, loco‐regional failure, overall survival	CT	298	Multiple institutes open dataset	CNN	AUC = 0.91 (distant metastasis), 0.78 (loco‐regional failure), 0.70 (overall survival); improved AUC = 0.83 (overall survival)
	31	Head and Neck Squamous Cell Carcinoma	Death prognosis and cancer recurrence rate prediction	CT	188	Multiple institutes open dataset	DL‐ANN	AUC = 0.934 and 0.932 for death prognosis and cancer recurrence Improved AUC = 0.947 and 0.956 for death prognosis and cancer recurrence respectively using GTV features
	33	Head and Neck Cancer	Two‐year overall survival and HPV status prediction	CT	1174	6 Institutes	GLM and GLORE	Comparable ROC between centralized and distributed models.
	32	Oropharyngeal Cancer	All‐cause mortality (ACM), local failure, distant metastasis (DM)	PET/CT	174	2 Institutes	Multiparameter LR	AUC = 0.65 for ACM, 0.73 for LF, 0.66 for DM; validation on independent dataset showed AUC = 0.68 for LF, 0.60 for ACM, and 0.65 for DM

Abbreviations: AdaBoost, adaptive boosting; ANN, artificial neural networks; BP, backpropagation; CoxBoost, a Cox proportional hazards model applying boosting techniques; CoxPH, Cox proportional hazards model; CNN, convolutional neural network; DL, deep learning; DeepSurv, a deep learning model adapted from Cox proportional hazards regression; DT, decision tree; GA, genetic algorithm; GLORE, generalized logistic regression; GLM, generalized linear models; glmboost, Gradient Boosting with Component‐wise Linear Models; glmnet, LASSO and Elastic‐Net regularized generalized linear model; KNN, k‐nearest neighbor algorithm; KSVM, kernel support vector machine; LASSO, least absolute shrinkage and selection operator; LGBM, Light Gradient Boosting Machine; LR, logistic regression; MergeNet, a neural network architecture; ML, machine learning; N‐MLTR, Neural Multi‐Task Learning for Regression; PGA, probabilistic genetic algorithm; PreSANet, Predictive Sparse Attention Network; ResNet, residual network; RF, random forest; RSF, random survival forest; Se‐ResNet, Squeeze‐and‐Excitation Residual Network; SE‐ResNeXt, Squeeze‐and‐Excitation ResNeXt, a convolutional neural network; SFBS, sequential backward floating selection; SHAP, SHapley Additive exPlanations; ST, survival tree; SVM, support vector machine; XGBoost, eXtreme Gradient Boosting.

While the models exhibit good performance in their specialized areas, one of the challenges in ML applications in healthcare is the creation of comprehensive models that can predict multiple outcomes simultaneously. Current models often operate in silos, predicting each outcome separately. Multitask learning is one approach that allows simultaneous prediction of several outcomes, potentially improving model performance through shared representation learning.

The methods used in these studies are diverse, featuring advanced techniques like 3D ResNet, cox proportional hazards (CoxPH), preSANet with pseudo‐volumetric CNN, generalized linear models (GLM), and generalized logistic regression (GLORE). Each of these methods brings strengths to different aspects of outcome prediction. For example, 3D ResNet and pseudo‐volumetric CNNs are adept at handling volumetric data, potentially improving the accuracy of survival and recurrence predictions. On the other hand, CoxPH models are traditionally strong for survival analysis, and GLM and GLORE can effectively handle binary outcomes such as the occurrence of toxicity.

Ultrasound imaging is primarily utilized for soft tissue evaluation (e.g., breast, prostate, and parotid gland), making it a valuable modality for certain studies focused on toxicity prediction, such as parotid injury in the head and neck region by Yang et al. in 2012 and 2014.^57^, ^58^, ^59^ However, it is worth noting that the studies incorporating ultrasound for toxicity prediction predominantly rely on radiomics techniques rather than ML methods, which were not included in our collection of studies.

### 4.3 Chest

**FIGURE 5:**
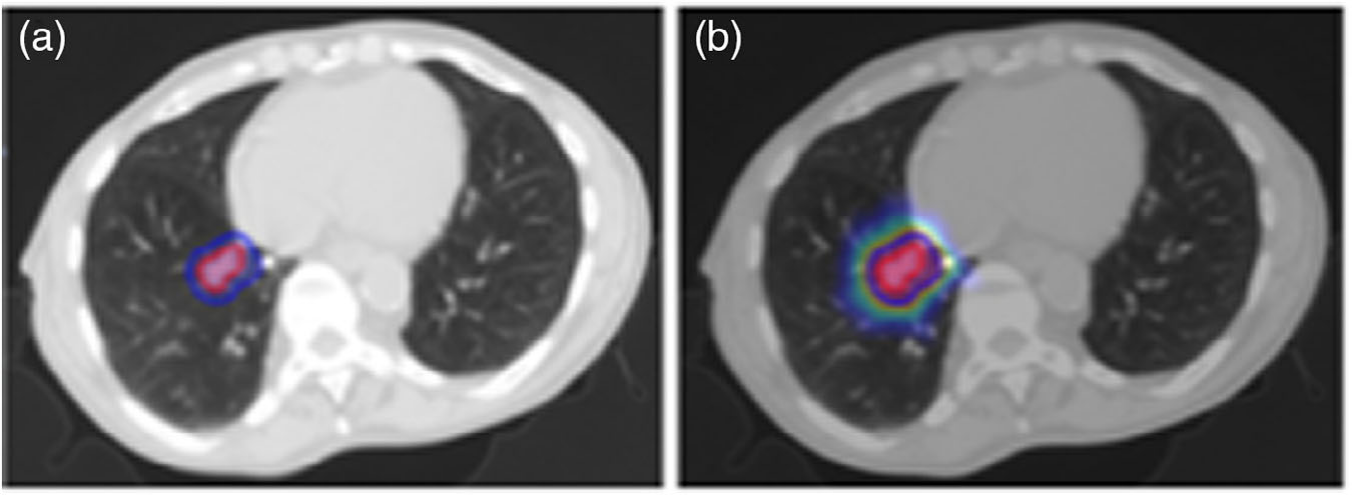
A 66‐year‐old male with Stage IA2 (cT1bN0Mx) adenocarcinoma in the right lower lobe of the lung. Images procured from our institution. (a) Axial view of the planning CT displaying CTV and PTV contours. (b) Axial view of the planning CT with CTV and PTV contours and a dose color wash, with a dose window level of 20 to 50 Gy.

The exploration of chest‐origin cancers has led to 25 studies, with a focus primarily on non‐small cell lung cancer. Figure 5 showcases the CT image with tumor contour and dose wash of a patient with adenocarcinoma (a subtype of non‐small cell lung cancer). Among these, 18 studies investigated survival outcomes, and 3 looked at recurrence outcomes. Additionally, out of 6 studies on toxicity, 4 highlighted the occurrence of radiation pneumonitis (Kapoor et al., 2023^60^; Niu et al., 2023^61^; Z. Zhang et al., 2023^62^; Cui et al., 2021^63^). Three of these studies utilized DL models, achieving AUC scores ranging from 0.729 to 0.91. In contrast, the study by Niu et al. (2023)^61^ employing Cox regression analysis reported AUC scores between 0.92 and 0.94.

Breast and lung cancers are both prevalent in the chest region, yet the studies included in Table 3 predominantly focused on lung cancer, with three studies centering on Esophageal Squamous Cell Cancer.^64^, ^65^, ^72^ In the context of breast cancer, there is a notable trend where studies primarily emphasize early detection, risk assessment, and staging rather than leveraging imaging data for toxicity prediction. This emphasis can be traced back to the types of data commonly utilized in these studies. Since predicting toxicity often necessitates intricate imaging details such as evolving tissue density or structural changes over time, researchers in breast cancer have predominantly employed conventional radiomics methods. While these methods offer valuable insights, they may not fully conform to the ML paradigms delineated earlier in our introduction. This disparity in data types and analytical approaches may illuminate the relatively limited utilization of ML specifically for toxicity prediction in breast cancer research compared to its application in other areas such as lung cancer.

**TABLE 3:** Survey of chest cancers.

Outcome	Reference	Tumor type	Category	Image modality	Sample size	Database source	Major method	Prediction performance
Survival	64	Esophageal Squamous Cell Carcinoma	Overall survival	PET/CT	142	1 Institute	LASSO Cox regression	c‐index = 0.74, 0.82, and 0.92 for CT‐based, PET‐based and dosiomics‐based in external validation
	65	Esophageal Squamous Cell Carcinoma	Progression‐free survival (PFS) and overall survival (OS)	CT	44	1 Institute	RF, RR, NB, SVM and ANN	The PFS and OS in the high‐prediction score group were significantly longer than that in the low‐prediction score group in RF model
	66	Small Cell Lung Cancer	Overall survival	Not Specified	21,093	Multiple institutes open dataset	DeepSurv	c‐index 0.7181 (train), 0.7208 (test).
	67	Primary (stage I‐IV) or recurrent lung cancer	Survival	CT	1151	1 Institute	Multitask deep neural network	c‐index = 0.68
	68	Non‐Small Cell Lung Carcinoma	1, 3, 5, 7‐year survival	CT	394	Multiple institutes open dataset	DT, BT, RF, SVM, GLM, and DL‐ANNs	Best AUC = 0.974 (RF) with 3‐year survival
	69	Stage I‐IIIA Non‐Small Cell Lung Cancer	Survival	CT	498	1 Institute	3D CNN	AUC 0.76 (UMCG), 0.64 (Maastro)
	70	Early‐stage non‐small cell lung cancer	2‐year survival	PET/CT	117	7 Institutes	SVM with LASSO	AUC = 0.77 (0.63–0.85)
	71	Locally Advanced Non‐Small Cell Lung Cancer	3‐year overall survival	CT	298	1 Institute	SVM	Accuracy = 92.50% (training) and 85.71% (validation). AUC = 0.965 (training) and 0.869 (validation)
	72	Esophageal Squamous Cell Cancer	Local recurrence‐free survival, and overall survival	CECT and clinical factors	397	3 Institutes	3D‐DenseNet and CoxPH regression	Best c‐index = 0.73 (training), 0.72 (validation)
	73	Non‐Small Cell Lung Cancer	Overall survival	PET/CT	2840	1 Institute	CoxPH regression	Found evidence that high muscle quantity is associated with better OS when muscle radiodensity is higher,
	74	Advanced Non‐Small Cell Lung Cancer	Survival	CT	100	1 Institute	LR, SVM Adboosting	Best AUC = 0.797 for LR with gini‐index feature selection
	75	Non‐Small Cell Lung Cancer	Overall survival, relapse‐free survival	Immunohistochemisty	151	1 Institute	EfficientUnet and ResNet	AUC for OS and RFS = 0.9 (internal), 0.87 (external)
	76	Non‐Small Cell Lung Cancer	Local recurrence‐free survival, disease‐free survival, overall survival	CT	135	1 Institute	DLPM	AUC for LRFS = 0.72, DFS 0.70, OS 0.66
	77	Non‐Small Cell Lung Cancer	Overall survival	4D‐CT	153	1 Institute	ANN	Test accuracy = 64.7%, associations with overall survival
	78	Non‐Small Cell Lung Cancer	2‐years overall survival	PET/CT	30	1 Institute	Linear SVM	AUC = 0.98 for sequential and 0.93 for concurrent chemoradiotherapy treatment groups
	79	Stage IV Adenocarcinoma Non‐Small Cell Lung Cancer	Overall survival	CT	195	Multiple institutes open dataset	Cox regression	Prognostic Index with c‐index = 0.576
	80	Non‐Small Cell Lung Cancer	2‐year overall survival	CT	1194	Multiple institutes	3D CNN	AUC = 0.70 for radiotherapy patients, AUC = 0.71 for surgery patients
Recurrence	81	Non‐Small Cell Lung Cancer	Probability of remission post‐chemotherapy and radiotherapy	CT	245	4 Institutes	Federated Learning (FL) with CNNs	AUC = 0.718 with centralized DL model, AUC = 0.725 with FL in simulation environment, AUC = 0.698 in real‐world FL model
	82	Locally advanced esophagus cancer	Pathological complete response	PET/CT and patient characteristics	98	1 Institute	CNN	Best accuracy = 0.81 and AUC = 0.83
Toxicity	60	Primary Lung Cancer	Radiation pneumonitis occurrence	CT and radiation dose datasets.	193	1 Institute	DenseNet‐121 and ResNet‐50	Best AUC = 0.91 with 3D DenseNet‐121 for three class
	61	Lung Cancer	Radiation pneumonitis occurrence	CT and dosimetric features	199	2 Institutes	LASSO Cox regression	Best AUC = 0.94 (testing set), 0.92 (validation set) with combined features
	62	Lung Cancer	Radiation pneumonitis occurrence	CT and radiation dose images	601	1 Institute	3D ResNet	Best performance AUC 0.83, accuracy = 0.82 (test‐set‐1)
	83	Non‐Small Cell Lung Cancer	Radiation‐induced lung injury, oncologic outcome	CT	181	1 Institute	Coxnet, Gradient Boost	c‐index 0.71–0.79 (lung fibrosis prediction)
Combined	63	Non‐Small Cell Lung Cancer	Radiation pneumonitis and local control prediction	PET, dosimetric data	469	1 Institute and multiple institutes open dataset	ADNN	c‐index = 0.660–0.727, AU‐FROC = 0.729
	84	Stage I/IIa Non‐Small Cell Lung Cancer	Local control, disease‐free survival, overall survival, and development of local lung injury up to fibrosis	CT	110	1 Institute	Cox regression and multivariate LASSO	Significant impact of continuous scores on all endpoints

Abbreviations: AdaBoost, adaptive boosting; ADNN, actuarial deep learning neural network; ANN, artificial neural network; BT, boosted tree; CoxPH, Cox proportional hazards model; Coxnet, a regularization technique for Cox proportional hazards models; CNN, convolutional neural network; DeepSurv, a deep learning model adapted from Cox proportional hazards regression; DenseNet, densely connected convolutional network; DL, deep learning; DLPM, Deep learning prognostication model; DT, decision tree; EfficientUnet, an adaptation of the U‐Net architecture that incorporates EfficientNet as the backbone; GLM, generalized linear models; LASSO, least absolute shrinkage and selection operator; LR, logistic regression; NB, Naive Bayes; ResNet, residual network; RF, random forest; RR, Ridge Regression; SVM, support vector machine.

Each study has distinct features and merits. For example, Niu et al. (2023)^61^ achieved the highest AUC by employing standardized pre‐intensity‐modulated radiation therapy CT images, radiation treatment planning, and clinical data from patients, using statistical methods for optimal feature selection to construct their models. Kapoor et al. (2023)^60^ addressed data imbalance and scarcity through minority class oversampling and data augmentation, developing models to classify different grades of radiation pneumonitis, and showcasing a novel approach.

Although their AUC performance is numerically lower, the other two studies^62^, ^63^ incorporated larger datasets and diverse methodologies. One study integrated multiomics data, including PET radiomics, cytokines, and miRNAs (Cui et al., 2021),^63^ while another combined CT and radiation dose images (Z. Zhang et al., 2023)^62^ (Table 3). These variations in input data and methodologies illustrate the complexity of predicting lung radiation pneumonitis, emphasizing the significant impact of input data choice on the model outcomes.

The current landscape in this research area demonstrates that there is no one‐size‐fits‐all approach. The selection of input data and modeling techniques can greatly affect the model's performance and applicability. This diversity reflects the ongoing challenge in the field: determining the most effective inputs and models for predicting outcomes, underscoring the intricate nature of ML and DL applications in medical research.

### 4.4 Abdomen and pelvis

While abdominal and pelvic cancers encompass a broad spectrum, to our knowledge, only 17 studies focus on these areas with 8 of them in survival, 4 in recurrence, and 5 in toxicity (Table 4).

**TABLE 4:** Survey of abdomen and pelvis cancers.

Outcome	Reference	Tumor type	Category	Image modality	Sample size	Database source	Major method	Prediction performance
Survival	88	Locally Advanced Cervical Cancer	Progression‐free survival (PFS)	PET/CT	190	2 Institutes	CoxPH and LASSO	Best AUC = 0.775, 0.767 for 3‐, and 5‐year PFS. Best c‐index = 0.724, 0.722 for 3‐, and 5‐year PFS
	89	Locally Advanced Cervical Cancer	Disease‐free survival (DFS) and overall survival (OS)	MRI	700	2 Institutes	RSF and GBM	RSF achieved AUC = 0.829, 0.809, 0.841 (test) for 1‐, 3‐, and 5‐year DFS, GBM achieved AUC = 0.904, 0.860, 0.905 (test) for 1‐, 3‐, and 5‐year OS
	90	Prostate Cancer	6‐year progression‐free survival	CT	64	1 Institute	LR with LASSO	Mean AUC = 0.76 (training) and 0.71 (testing). Accuracy = 0.778 (training) and 0.842(testing)
	91	Primary Gastrointestinal Lymphoma	Survival	Not Specified	11250	3 Institutes and multiple institutes open dataset	DeepSurv, RSF and CoxPH	Best performance is DeepSurv, c‐index = 0.760 (training), 0.742 (test), 0.707 (external validation)
	92	Pancreatic Ductal Adenocarcinoma	Survival	CT	1516	5 Institutes	ConvLSTM and CNN	HR for high vs. low risk in validation cohorts = 2.03 and 2.47
	93	Gallbladder Cancer.	Survival	CT	195	2 Institutes	3D‐DenseNet network	c‐index = 0.787; AUC = 0.827, 0.865, and 0.926 for 1‐, 3‐, and 5‐year survival.
	94	Colorectal Liver Metastases	Survival	CT	97	Multiple institutes open dataset	RSF	Highest average prediction accuracy occurred when combining both radiomics of the liver parenchyma and tumor volume with treatment data (c‐index = 0.73. Utilizing only radiomic data from the liver parenchyma and tumor volume resulted in a c‐index = 0.68
	95	Gastric cancer	Survival	Not Specified	1190	Multiple institutes	SRN	Mean AUC = 0.92 at the fifth year
Recurrence	96	Locally Advanced Cervical Cancer	Local failure	MRI and PET	189	3 Institutes	RF, SVM and Multivariate regression	Success in harmonizing features of new patients from a known center
	97	Cervical Cancer	Recurrence within the irradiation field	MRI	87	1 Institute	RF	AUC‐ROCs = 0.82, 0.82, and 0.86 for expanded VOI models
	98	Cervical Cancer	Local and distant failures	PET/CT	142	1 Institute	NiN	Accuracy = 89% for local recurrence, 87% for distant metastasis.
	85	T2‐4 N0‐1 rectal adenocarcinoma	Complete response	CT	95	3 Institutes	DNN, LR and SVM	Accuracy = 80% for DNN, 69.5% for LR, 71.58% for SVM
Toxicity	99	Prostate Cancer	Rectal toxicity	CT and MR with dosimetric, and clinical variables	70	1 Institute	RF, DT, LR, and KNN	Best performance is with MRI: AUC = 0.79, Accuracy = 77.75%
	100	Rectal Cancer	Hematologic toxicity	CT	336	1 Institute	Hybrid machine learning model	Accuracy = 0.843 (discovery), 0.802 (validation)
	101	Prostate Cancer	Acute and sub‐acute genitourinary toxicities	CT	50	1 Institute	Delta‐radiomic features	AUC = 0.83 for IPSS; over 0.7 for CTCAE grades
	102	Primary Liver Cancer.	Post‐radiotherapy hepatobiliary toxicities	CT	122	1 Institute	CNN	Accuracy = 0.73 for hepatobiliary toxicity prediction
	103	Liver Cancer	Hepatobiliary toxicity	CT	125	1 Institute	CNN	AUC = 0.79 for CNN alone, AUC = 0.85 for combined CNN and neural network analysis

Abbreviations: ConvLSTM, convolutional long short‐term memory; CoxPH, Cox proportional hazards model; CNN, convolutional neural network; DeepSurv, a deep learning model adapted from Cox proportional hazards regression; DenseNet, densely connected convolutional network; DNN, deep neural network; DT, decision tree; GBM, Gradient Boosting Machine; KNN, k‐nearest neighbor algorithm; LASSO, least absolute shrinkage and selection operator; LR, logistic regression; NiN, Network in Network; RF, random forest; RSF, random survival forest; SRN, Stochastic Residual Network; SVM, support vector machine.

**FIGURE 6:**
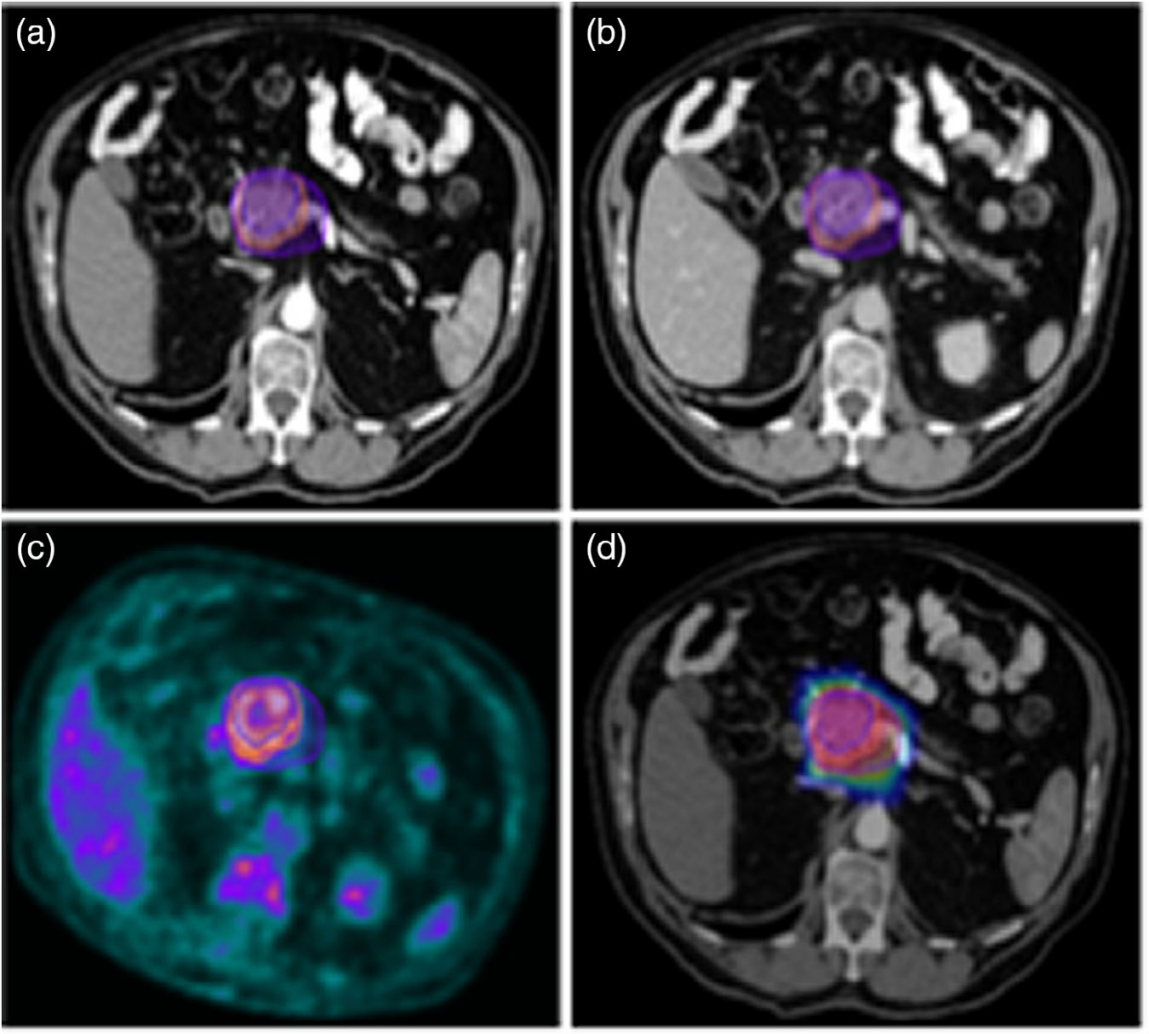
A 76‐year‐old male with borderline resectable adenocarcinoma of the pancreatic head. Images procured from our institution. (a) Axial view of the planning CT in arterial phase showing gross tumor volume (GTV) and planning target volumes (PTV 4000 and PTV 3300) contours. (b) Axial view of the planning CT in venous phase with contrast highlighting GTV, PTV 4000, and PTV 3300. (c) axial view of the PET scan with GTV, PTV 4000, and PTV 3300 contours, window level 0 to 6 SUV. (d) Axial view of the planning CT in arterial phase with GTV, PTV 4000, and PTV 3300 contours, plus radiation therapy (RT) plan dose color wash, dose window level 20 to 40 Gy.

For abdominal cancers, research is primarily directed at pancreatic ductal adenocarcinoma, primary gastrointestinal lymphoma, as well as gallbladder and gastric cancers, all concentrating on survival outcomes. Investigations also delve into colorectal liver metastases and various forms of liver cancer, evaluating both survival and post‐radiotherapy hepatobiliary toxicities. For instance, Figure 6 presents imaging from a patient diagnosed with borderline resectable adenocarcinoma of the pancreatic head. These images then can be used individually or in combination as the input for the model training.

**FIGURE 7:**
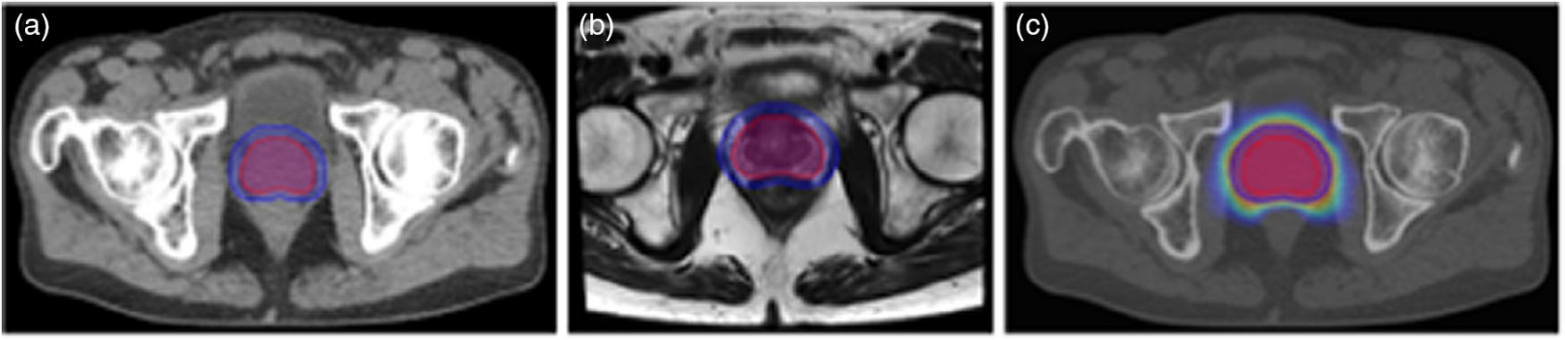
Imaging of a 65‐year‐old male with intermediate‐risk (Stage T1c) adenocarcinoma of the prostate procured from our institution. (a) Axial view of the planning CT with GTV and PTV outlined. (b) Axial T2‐weighted MRI showing GTV and PTV. (c) Axial view of the planning CT with GTV and PTV contours, plus a dose color wash; dose window level from 35 to 70 Gy.

In the pelvic region, studies predominantly feature prostate and cervical cancers. Research concerning prostate cancer spans several areas of interest, including rectal toxicity, progression‐free survival, and genitourinary toxicities. Cervical cancer studies, on the other hand, assess local failure rates and recurrence, both within and outside the irradiation field. Additionally, a study focuses on rectal adenocarcinoma (Bibault et al., 2018),^85^ specifically its complete response rate to treatment. Figure 7 depicts imaging of a 65‐year‐old male with intermediate‐risk adenocarcinoma of the prostate.

While certain studies have explored abdominal and pelvic cancers, their focus has primarily been on diagnosis, early detection, or staging purposes. For instance, Wang et al. (2022)^86^ and Feng et al. (2017)^87^ conducted studies on prostate cancer prediction using ultrasound. However, the existing studies that utilized medical imaging for outcome prediction are quite limited, either due to methodological restrictions or their specific research goals, highlighting the need for more comprehensive investigations in this area. Each study collected in Table 4 provides insights into clinical value to patients, the scant number of investigations for each cancer type poses challenges. It complicates the task of effective comparison and constrains our capacity to extrapolate these findings into broader contexts. Consequently, the development of generalizable and robust predictive models for widespread clinical application remains a challenge.

## 5 DISCUSSION

### 5.1 Summary

When looking at study preferences across all treatment sites, over half of the research prioritizes survival‐related outcomes. This emphasis highlights the critical importance of prognosis in managing these cancers, which often involve complex treatments and vary significantly in patient outcomes. Survival analysis plays a key role in cancer research, offering insights into how effective treatments are and helping guide clinical decisions.

Using predictive modeling for treatment‐related toxicity prediction marks a growing area for DL. These models aim not only to predict patient survival but also to foresee possible treatment complications. For instance, radiation pneumonitis, a significant concern in lung cancer treatment, was a focus in 4 of 6 toxicity studies. This increased attention to lung damage from radiation shows a rising concern for lung cancer patients' quality of life, stressing the need to balance treatment effectiveness with minimizing adverse effects. Predictive toxicity modeling is complex as it requires analyzing the tumor and surrounding healthy tissue that radiation might affect. The DL models used in these studies are designed to identify subtle imaging patterns indicating a higher risk of toxicity. These models are valuable tools for physicians and can help tailor radiation doses to optimize treatment outcomes while reducing the risk of organ damage.

In the area of abdominal and pelvic cancers, despite the innovative use of ML to predict treatment outcomes, there are comparatively fewer studies. This gap could be due to several reasons. First, cancers in the abdominal and pancreatic areas have historically received less attention than more prevalent cancers like lung or brain cancer. This may be related to public health priorities, the prevalence of these cancers, or the novelty of certain cancer types in drawing research interest. As a result, these cancers have been studied less, leading to less existing research literature. Second, data availability in these areas also poses challenges, largely because there are relatively fewer public datasets available for these body parts. This scarcity limits opportunities for broader research and application of ML techniques. Furthermore, the small bowel in the abdominal region is particularly radiosensitive, and irradiation of the abdominal region is therefore reserved for specific cancer types such as prostate and rectal cancer, which further limits the applicability of ML in this area.^104^ Lastly, the technical challenges of imaging and analyzing these areas pose a unique challenge since abdominal and pancreatic tissues are difficult to image due to their location, density, and the proximity of other organs. This difficulty in generating consistent, high‐quality imaging data impacts the development of machine‐learning models that depend on such data.

### 5.2 Overview of trend

This review of cancer radiotherapy outcome prediction, integrating ML techniques, highlights key advancements and challenges. We categorized studies by treatment sites and observed significant trends, including a linear increase in publications since 2020 (Figure 1a). This surge corresponds with advancements in DL, leading to refined predictive models.

Our analysis, particularly on survival (Figure 1b), aligns with oncology's goals to extend lifespans and enhance post‐treatment quality of life. It also emphasizes the importance of managing toxicity and preventing cancer recurrence, key elements in patient‐centric treatment approaches. Nonetheless, our review discerned a potential skew deriving from an underrepresentation of studies concentrating on toxicity. This bias may be attributed to the complexities associated with toxicity, a multifaceted term encompassing a spectrum of adverse effects induced by radiotherapy, and the constraints of the keywords used in our search methodology. Toxicity can manifest in various forms, such as hematological complications, dermatitis, or lymphedema, and its primary manifestations differ based on cancer type, location, and stage, among other factors. For instance, radiation pneumonitis is a prevalent toxicity in lung cancer treatments, while dry mouth, often due to parotid gland irradiation, frequently accompanies head‐and‐neck cancer therapies. Although there exists a subset of research focusing on specific toxicities and employing ML to predict outcomes, these studies were not captured in our literature search due to the keyword constraints. Consequently, this review encapsulates a limited selection of toxicity‐related studies, reflecting an inherent bias in outcome prediction literature that predominantly values survival and recurrence metrics.

Ultrasound imaging, despite being a non‐invasive and relatively low‐cost option, is less frequently employed in outcome prediction. Ultrasound images generally have a smaller image field than CT and MRI, making them less effective in capturing the detailed cross‐sectional views needed for precise tumor delineation and anatomical location. Only limited studies have explored the utilization of ultrasound‐based radiomics features for assessing radiation‐induced toxicity on breast^105^ or parotid glands after radiotherapy.^57^, ^106^ Additionally, ultrasound is highly operator‐dependent and can yield variable results based on the technician's skill level. This introduces an element of subjectivity less prevalent in CT or MRI imaging. Thirdly, ultrasound has limitations in in‐depth penetration, particularly in oncology, where tumors may be deeply seated or obscured by bone or gas‐filled organs. This constraint restricts its utility in the visualization of internal structures and in accurately assessing the tumor's size and extent. These limitations are fundamental when considering the input data required for reliable outcome prediction, leading to ultrasound's limited adoption in this area compared to other imaging.

Furthermore, several studies^15^, ^72^, ^82^, ^99^ have embraced a more integrative approach, combining imaging modalities with clinical data. Although models that incorporate both images and patient data are inherently more complex, they offer a comprehensive view, merging the anatomical precision of imaging with the contextual richness of clinical information. This implementation can unearth small correlations and patterns that are indiscernible through a single type of data, thereby furnishing a more robust foundation for ML algorithms. Such models can accommodate a broader spectrum of variables, from genetic markers to environmental factors, thereby providing a holistic patient profile that could revolutionize personalized treatment pathways. Incorporating both imaging and clinical data into ML models presents a series of challenges that extend beyond the complexity of model construction. The amalgamation of high‐dimensional imaging data with varied clinical information necessitates advanced data processing capabilities and sophisticated algorithms that can handle such complexity. Ensuring the integrity and compatibility of multimodal data is also a significant hurdle. Datasets must be meticulously curated and harmonized to enable the integration of disparate data types, such as standardized imaging formats with heterogeneous clinical records.

The diversity of data sources depicted in Figure 1d presents a critical dimension of current research in cancer radiotherapy. With over half of studies drawing from single‐institution datasets, the potential for nuanced and diverse data interpretation is limited. This reliance on homogeneous data pools could skew predictive models, leading to outcomes that may not be universally applicable. This is of particular concern regarding underrepresented minority populations that have historically been misrepresented in medical treatment algorithms. The remaining 47% of studies utilizing multi‐institutional datasets mark a crucial step towards overcoming these limitations. Such collaborative efforts are not only a testament to the collective push for advancement in the field but also essential in enhancing the robustness of predictive models. By pooling data from a wider array of patient demographics and treatment responses, these models can achieve a level of sophistication and generalizability more reflective of the diverse patient populations they aim to serve. This concerted effort towards data diversity is instrumental in developing ML models that can deliver precise and reliable outcome predictions across various clinical settings.

### 5.3 Challenge of data

The pursuit of comprehensive data collection for outcome prediction in cancer radiotherapy faces significant barriers. The outstanding challenge is the requirement for protracted patient follow‐up, which is a resource‐intensive task. For example, monitoring 2‐year or even 5‐year survival rates necessitates continuous patient engagement. The continuity of tracking is frequently compromised by factors such as patients transitioning between treatment centers, relocating, or simply becoming lost to follow‐up, which introduces substantial logistical difficulties and sets extra demands on healthcare professionals to maintain consistent data collection amidst a complex landscape of patient movements.

In ML applications in radiotherapy, cross‐institutional cooperation is crucial to overcome data limitations. However, this collaboration introduces its complexities, notably regarding data privacy and standardization. Privacy concerns are paramount as patient data is subject to stringent confidentiality regulations, making sharing information across institutions a sensitive process. Additionally, data standardization emerges as a significant barrier. Institutions often use varied data recording practices, leading to heterogeneous datasets that are difficult to merge and analyze consistently. This lack of standardization can skew model training and validation, potentially leading to biased or inaccurate predictive outcomes.

Image harmonization techniques are developing as a promising solution to address these challenges. Image harmonization aims to reduce variability in imaging data that originates from different sources, scanners, or imaging protocols. By applying algorithms that adjust for these variances, researchers can create harmonized datasets that are more suitable for collective analysis and can be pooled from diverse institutions without compromising data integrity. This harmonization is critical when considering the subtle nuances that DL models must learn to accurately predict treatment outcomes.

The development of standardized protocols for data collection and image processing, along with robust encryption and anonymization methods, will be pivotal in advancing cross‐institutional studies. These efforts will enable the aggregation of large‐scale, multi‐source datasets necessary for training sophisticated deep‐learning models. As a result, such models will be able to provide more accurate, generalizable, and clinically relevant insights, driving progress in personalized radiotherapy treatments.

The advancement of DL in cancer radiotherapy outcome prediction is a journey fraught with obstacles, yet one defined by the relentless pursuit of innovation and boundless potential. The adoption of image harmonization and improved data standardization practices represents a forward leap in addressing the current limitations, setting the stage for a future where data‐driven insights can be leveraged to deliver precision medicine and enhance patient care.

### 5.4 Interpretability

The combination of traditional ML and DL methods have become increasingly prevalent. While traditional ML models are generally recognized for their interpretability, DL models often present challenges. Interpretability is crucial for a ML model, as it involves making the model's internal processing understandable to humans. Traditional algorithms, although sometimes described as “black boxes”, have been less complex and more interpretable than their deep‐learning counterparts.

Interpretability is vital, especially in critical fields like medical diagnosis and treatment planning. Stakeholders, including medical professionals and regulatory agencies, require a clear understanding of the basis on which models make predictions. This requires building trust and ensuring the reliability and safety of the technology applied to patient care. The natural hesitation to depend on complex and seemingly mysterious technology can hinder the adoption of these advanced models in clinical settings.

DL models, known for their layered and intricate computational processes, pose significant challenges in interpretability. These models typically involve complex convolutional processes and multiple layers of computation, making their internal workings less transparent and more difficult to understand. This lack of clarity in understanding how DL models process data and make decisions can be a significant barrier, especially in outcome prediction, where the rationale behind each prediction is as crucial as the prediction itself.

**FIGURE 8:**
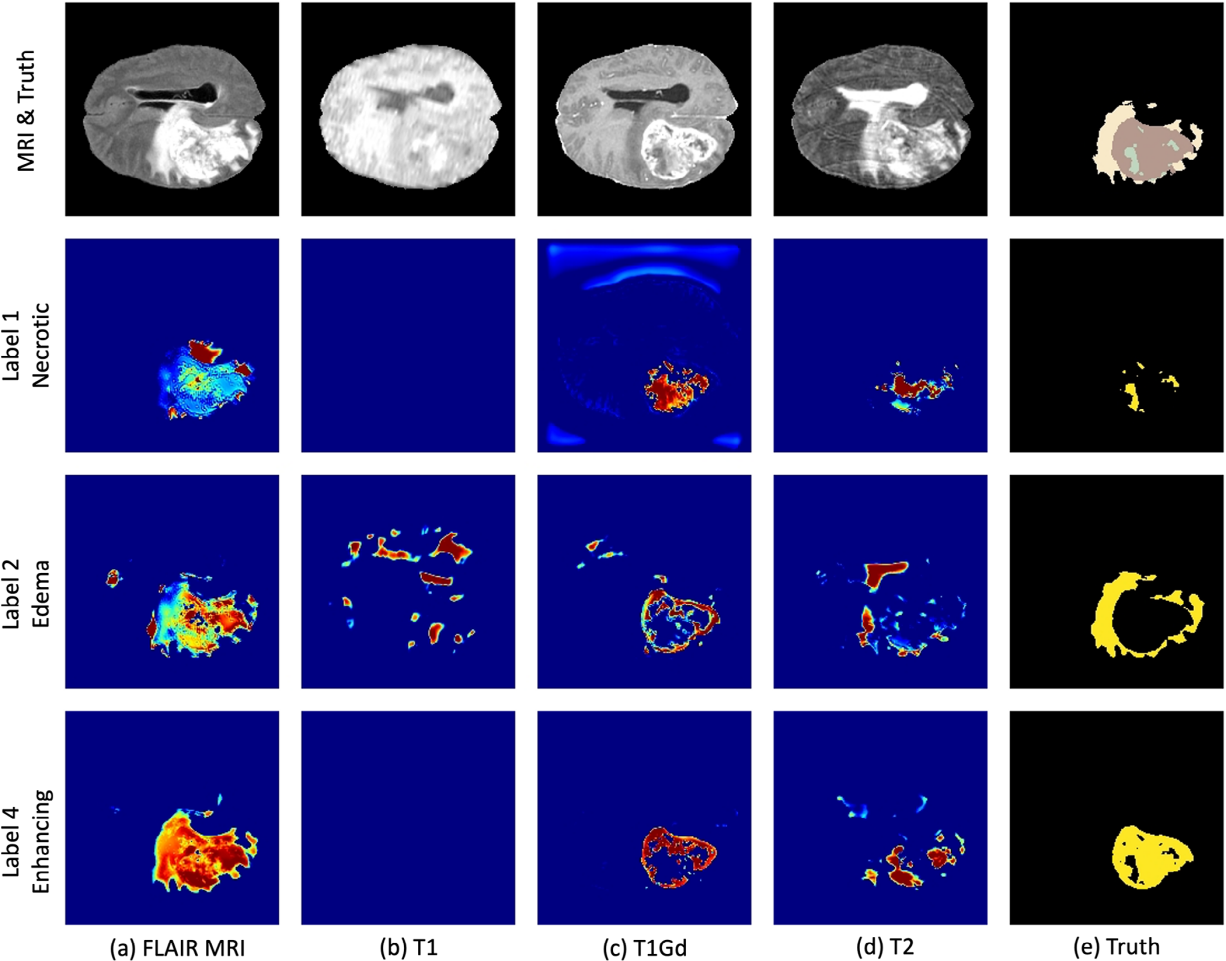
Impact of MRI input modality in the detection of different tumor labels. The first row shows the input MRI sequences and the ground truth annotations. The following rows correspond to label 1 (the necrotic tumor core), label 2 (the peritumoral edema), and label 4 (the enhancing tumor). In the saliency maps, warmer regions represent a high score for the specified label detection. (*Source*: Image from Zeineldin et al., 2024^107^).

In response to the challenges of understanding complex DL models, significant progress is being made toward enhancing their interpretability. Researchers are innovating with techniques like probability maps to visualize and decode the data processing across various layers of these models. Such initiatives are integral to a larger effort to clarify the operations of DL systems, thereby increasing their transparency and accessibility. For example, the study by Zeineldin et al. (2024)^107^ shown in Figure 8 generates heatmaps that color‐code the importance of various regions on the input images, helping to interpret the rationale behind the model's decision‐making process for each case. By occluding parts of the image and observing changes in the model's output probability, researchers can identify which areas of the image are most critical for the model's prediction. Such methods are precious in medical imaging, where understanding the basis of a model's decision can provide insights into its reliability, improve trust in its predictions among clinicians, and potentially reveal new patterns or features associated with specific outcomes. This push for greater interpretability is vital in medical applications, where grasping the logic behind a model's predictions is indispensable for gaining clinical trust and meeting regulatory standards. Furthermore, clinicians must be well‐equipped to explain the rationale of ML outputs to patients. By advancing these interpretability methods, the aim is to render DL models not just potent in their predictive accuracy but also reliable and intuitive for practitioners, thereby improving patient care and the accuracy of outcome predictions.

## 6 CONCLUSION

This comprehensive review has elucidated the dynamic interplay of ML with cancer radiotherapy outcome prediction, charting the evolution of research trends and technological adoption. The gradual yet steady increase in publications post‐2020 attests to the burgeoning confidence in ML's potential within oncology. The spotlight on survival, toxicity, and recurrence in outcome prediction reflects a multifaceted clinical approach, marrying the quest for longevity with quality‐of‐life considerations.

Despite the abundance of CT scans being clinically favored for their precision, the integration of MRI and clinical data is gaining traction, promising more holistic patient profiles. However, the challenges of data collection, privacy concerns, and the need for model interpretability loom large, necessitating innovative solutions such as image harmonization and standardized data protocols.

In the less explored terrains of abdominal, rectal, and pancreatic cancers, the scarcity of studies is indicative of disproportionate research focus and data limitations. Broadening the scope of research to include these areas is crucial, as is fostering cross‐institutional collaborations to overcome data shortages and enhance model reliability.

As we forge ahead, the interplay of advanced ML models and traditional techniques must navigate the intricacies of interpretability and data privacy. The endgame remains steadfast—to leverage these sophisticated tools to revolutionize patient care, offering personalized, effective, and compassionate treatment pathways.

## AUTHOR CONTRIBUTIONS

Xiaohan Yuan: Made substantial contributions to the conception and design of the work, as well as to the acquisition, analysis, and interpretation of data. Revised the manuscript critically for important intellectual content. Chaoqiong Ma, Mingzhe Hu, Richard L.J. Qiu, Elahheh Salari, and Reema Martini: Made substantial contributions to the conception of interpretation of the data for the work. Revised the manuscript critically for important intellectual content. Xiaofeng Yang: Made substantial contributions to the conception of interpretation of the data for the work as well as design of the work. Revised the manuscript critically for important intellectual content. All authors agreed to be accountable for all aspects of the work in ensuring that questions related to the accuracy or integrity of any part of the work are appropriately investigated and resolved.

## CONFLICT OF INTEREST STATEMENT

The authors declare no conflicts of interest.

## INSTITUTIONAL REVIEW BOARD STATEMENT

The medical images used in this study, procured from our institute, were obtained under an Institutional Review Board (IRB) approved retrospective study and are compliant with the Health Insurance Portability and Accountability Act (HIPAA).
